# A Microarray-Based Analysis of Gametogenesis in Two Portuguese Populations of the European Clam *Ruditapes decussatus*

**DOI:** 10.1371/journal.pone.0092202

**Published:** 2014-03-18

**Authors:** Joana Teixeira de Sousa, Massimo Milan, Luca Bargelloni, Marianna Pauletto, Domitília Matias, Sandra Joaquim, Ana Margarete Matias, Virgile Quillien, Alexandra Leitão, Arnaud Huvet

**Affiliations:** 1 IFREMER, UMR CNRS 6539, Laboratoire des Sciences de l'Environnement Marin, Plouzané, France; 2 IPMA, Olhão, Portugal; 3 Department of Comparative Biomedicine and Food Science, University of Padova, Agripolis, Legnaro, Italy; Laboratoire de Biologie du Développement de Villefranche-sur-Mer, France

## Abstract

The European clam, *Ruditapes decussatus* is a species with a high commercial importance in Portugal and other Southern European countries. Its production is almost exclusively based on natural recruitment, which is subject to high annual fluctuations. Increased knowledge of the natural reproductive cycle of *R. decussatus* and its molecular mechanisms would be particularly important in providing new highly valuable genomic information for better understanding the regulation of reproduction in this economically important aquaculture species. In this study, the transcriptomic bases of *R. decussatus* reproduction have been analysed using a custom oligonucleotide microarray representing 51,678 assembled contigs. Microarray analyses were performed in four gonadal maturation stages from two different Portuguese wild populations, characterized by different responses to spawning induction when used as progenitors in hatchery. A comparison between the two populations elucidated a specific pathway involved in the recognition signals and binding between the oocyte and components of the sperm plasma membrane. We suggest that this pathway can explain part of the differences in terms of spawning induction success between the two populations. In addition, sexes and reproductive stages were compared and a correlation between mRNA levels and gonadal area was investigated. The lists of differentially expressed genes revealed that sex explains most of the variance in gonadal gene expression. Additionally, genes like *Foxl2*, *vitellogenin*, *condensing 2*, *mitotic apparatus protein p62*, *Cep57*, *sperm associated antigens 6, 16* and *17*, *motile sperm domain containing protein 2*, *sperm surface protein Sp17*, *sperm flagellar proteins 1 and 2* and *dpy-30*, were identified as being correlated with the gonad area and therefore supposedly with the number and/or the size of the gametes produced.

## Introduction

The European clam, *Ruditapes decussatus* (Linnaeus, 1758) is a bivalve mollusc of the family Veneridae, native to the European Atlantic and Mediterranean coastal waters. The European clam, despite a relatively low European production (8200 tons/year) [1], is considered a high value seafood product and one of the most important bivalve species economically in Southern European countries like Italy, Spain and Portugal [2].

The culture of *R. decussatus* is clearly limited by the availability of seed. Its production is almost exclusively based on natural recruitment, which is subject to high annual fluctuations due to pollution and other environmental factors. To address this situation, artificial spawning and larval rearing programs were developed to provide an alternative source of seed [2], but still need improvement to gain robustness of seed production.

*R. decussatus* is a gonochoric molluscan species (possessing separate sexes) that reproduces annually [3]. Generally, the gonad regresses at the end of the annual reproductive cycle, which is the end of summer in temperate regions, and regenerates at the beginning of the following one (the end of winter). During the initial stage of gametogenesis, period of sexual rest (I), gonadal follicles are absent and connective and muscular tissue occupies the entire zone from the digestive gland to the foot. There is no evidence of gonadal development and determination of sex is not possible. During the second stage of gametogenesis (II) follicles and gonadal acini begin to appear in females and males, respectively. They increase in size, and appear filled with oocytes in the growth phase in the females and with immature gametes (spermatogonia and spermatocytes) in the males. During advanced gametogenesis (III) the follicles occupy a large part of the visceral mass and it's possible to observe the first signs of partial emission of gametes when it occurs. The maturation period (IV) corresponds to the maturity of the majority of gametes. Throughout this period partial spawning may occur, and it concludes with the total emission of gametes [4].

Currently, the knowledge about the molecular mechanisms of reproduction in marine bivalves is very limited, but is expected to increase rapidly, due to the advent of high throughput genomic approaches [5]. Here, a large and exhaustive approach is proposed for investigating the molecular bases of variability in reproductive success and, more generally, the reproductive mechanisms in *R. decussatus*. Some specific studies have recently explored this topic. The gonadal transcriptome was described and lists of genes of interest for reproductive stage and sex were established for the alternative and irregular protandrous hermaphrodite, the Pacific oyster, *Crassostrea gigas* [5] and for the functional hermaphrodite scallop *Nodipecten subnodosus* [6]. Most importantly, cross-referencing these lists of genes allowed the identification of potential markers of early sex differentiation and new highly valuable information on genes specifically expressed by mature spermatozoids and mature oocytes. Additionally, dedicated studies have been made in *Argopectyen purpuratus*, *C. gigas* and *Mytilus galloprovincialis* emphasizing specific reproductive processes [7], [8], [9], [10], [11].

In *R. decussatus*, apart from a few specific gene expression analyses (see [12]) and a first version of DNA microarray designed to unravel host-parasite interactions [13], very little information is available on genome-wide expression profiling for physiological, environmental or aquaculture questions.

For the present work, cDNA libraries of oocytes, larval stages and different gonadal maturation stages were sequenced on the Illumina platform, and a custom oligonucleotide microarray representing 51,678 assembled contigs was designed and used to characterize the transcriptomic bases of reproduction in *R. decussatus*. Microarray analyses were performed in four gonadal maturation stages of two Portuguese wild populations, Ria Formosa in Southern Portugal (37°01′N; 07°49′W) and Ria de Aveiro in Western coast of Portugal (40°42′N; 08°40′W). These populations were already characterized by different responses to spawning induction, which is of great interest to study in a context of improvement of aquaculture production [2], [14].

The present study provides new highly valuable genomic information for the understanding of reproduction of this species and emphasizes some candidate genes as possible starting points for further functional studies, for instance on spawning and gamete quality with the aim of elucidating how and to what extent environmental factors affect relevant reproductive-gene expression.

## Materials and Methods

### Ethics statement

The European clam is not considered as an endangered or protected species in any Portuguese or international species catalog, including the CITES list (www.cites.org). The European clams from Ria de Aveiro (40°42′N; 08°40′W) and Ria Formosa (37°01′N; 07°49′W) were produced and captured with the permission of DGRM (Direção-Geral de Recursos Naturais, Segurança e Serviços Marítimos), APA (Agência Portuguesa do Ambiente) and PNRF (Parque Natural da Ria Formosa - National park defined by the Ramsar Convention).

### Animal sampling for RNA-sequencing

Clams were collected in Ria de Aveiro (40°42′N; 08°40′W), and conditioned in a common garden setting to accelerate their gonad development from October 2011 to January 2012 in the experimental bivalve hatchery of Portuguese Institute of Sea and Atmosphere (IPMA) in Tavira, Portugal. Food regimes consisted of different algal mixtures containing *Isochrysis galbana* (clone T-ISO) and *Skeletonema costatum* (Ria Formosa autochthone clone). Microalgae were cultured in 80 L bags with f/2 medium [15], in a temperature-controlled room at 20±2°C under continuous illumination (9900 lux) and aeration. Gonad samples were collected and immediately dissected: a transversal section of the gonadal area was fixed for histological examination and the rest of the gonads were frozen immediately in liquid nitrogen. Later, the gonads were crushed into a fine powder at −196 °C with a Dangoumau mill and stored in liquid nitrogen until RNA extraction. For the histological analysis, a 3-mm cross section of the visceral mass of each sample was excised using a microtome cutter in front of the pericardic region and immediately fixed in 10 to 20 ml modified Davidson's solution [16] at 4°C for 48 h. After, the modified Davidson's solution was discarded and 10 to 20 ml 70% ethanol was added and kept at 4°C.

Sections were dehydrated in ascending ethanol solutions, cleared with a xylene substitute named Microclearing (Diapath, Italy), and embedded in paraffin wax. Sections of 5 μm were cut, mounted on glass slides and stained with Harry's hematoxylin-Eosin Y [17]. Slides were then examined under a light microscope and sex and stages were determined according to the 4 stages previously described (see Introduction). Percentage areas of gonadic tubules, connective tissue and digestive gland were then determined on each histological section. Slides were scanned with a digital scanner (hp scanjet 7400 c) and the images saved in *.TIFF format. Tissue areas were then measured using image analysis software (Imaq Vision Builder, National Instruments Corp.). Gonad area percentage was estimated as pixel number, from gonad / pixel number on total sections, as described in [18]. 35 individuals corresponding to five gonad samples of each reproductive stage and sex were chosen for RNA-sequencing.

### Animal sampling for microarray analysis

**Figure 1:**
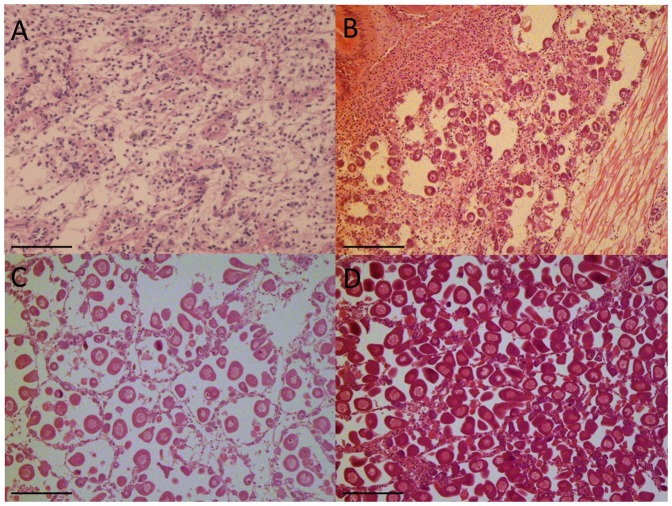
Photomicrographs showing stages in the development of *Ruditapes decussatus* female gonad. A. Sexual rest. B. Initiation of gametogenesis; C. Advanced gametogenesis; D. Maturation. Scale bar: 200 μm.

**Figure 2:**
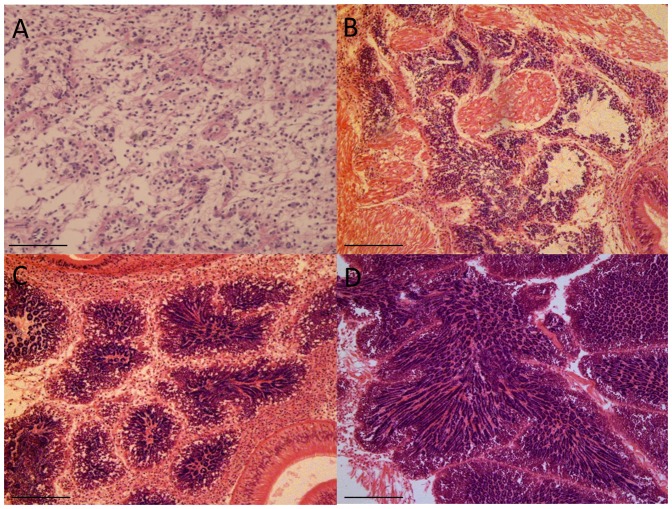
Photomicrographs showing stages in the development of *Ruditapes decussatus* male gonad. A. Sexual rest. B. Initiation of gametogenesis; C. Advanced gametogenesis; D. Maturation. Scale bar: 100 μm in A, B, C; 200 μm in D.

Twenty clams from two wild populations - Ria Formosa (South of Portugal) and Ria de Aveiro (Northern of Portugal) - were sampled monthly between July 2011 and July 2012. At each sampling date the gonads of the collected clams were immediately dissected for RNA extraction and a transversal section of the gonadal area fixed for histological analysis, as described above. Gene expression analysis was done on 64 individual gonads representative of all the reproductive stages and sexes from both populations, chosen on the basis of histological data (Figure 1; Figure 2; Table 1).

**Table 1:** Number and sex of the 64 individuals of each gonad developmental stage from the two studied populations used in the microarray analysis.

Gonad developmental stage	Sex	Ria Formosa	Ria de Aveiro
I		5	5
II	F	5	5
	M	5	5
III	F	5	5
	M	3	7
IV	F	5	3
	M	3	3

### RNA extraction

For RNA-sequencing and microarray analysis, total RNA was isolated using Extract-all (Eurobio) procedure. RNA quality and integrity were analysed on the Agilent bioanalyzer using RNA nanochips and Agilent RNA 6000 nanoreagents (Agilent Technologies, Waldbronn, Germany). RNA concentrations were measured at 260 nm using a ND-1000 spectrophotometer (Nanodrop Technologies) using the conversion factor 1 OD = 40 mg/mL total RNA. Samples were stored at −80°C until further use.

### cDNA library construction and sequencing

Sequencing of gonadal maturation stages was performed through a multiplexing strategy on the Illumina platform HiSeq2000. A total of 7 cDNA tagged libraries from gonads of 5 individuals, of each developmental stage (sexual rest stage I and stages II/III/IV in both sexes), were prepared on a 7-plex paired-end sequencing lane (2×100 bp). cDNA libraries were constructed, amplified and sequenced at BGI Tech (Shenzhen, China). All the Illumina reads were analyzed with FastaQC software in order to assess the sequence quality. Illumina reads have been deposited in GenBank (SRA) with the accession numbers SRR974930, SRR974931, SRR974932, SRR97493, SRR974934, SRR974935, SRR974936.

### Assembly and transcriptome annotation

To obtain a wider *R. decussatus* transcriptome representation, Roche 454 and Illumina reads have been added to the Illumina reads produced by a 7-plex paired-end sequencing lane (Table S1). Specifically, additional Illumina reads have been obtained through the sequencing of stripped and spawned oocytes, larvae at 48 hours post-fertilization, and larvae at 17, 21 and 30 days post-fertilization cDNA libraries (Pauletto et al. unpublished data; personal communication). In addition, reads obtained by the sequencing by 454 Roche of hemocytes, oocytes and larval stages cDNA libraries have been also considered for the assembly (Novoa et al., unpublished data; personal communication). These libraries have been produced within the European project REPROSEED and will be public with the complete transcriptome of *R. decussatus* (Pauletto et al, Novoa et al. unpublished data). All these data were merged also with publicly available Sanger sequences and nearly 500,000 reads obtained with 454 sequencing of larvae, gonads and several adult tissues (MGE011 and cDN18; SRA058431; [13]). A summary of the origin of *R. decussatus* sequences and assembly information are shown in Table S1.

Assembly was performed separately for each library by using MIRA3 or CLC Genomic Workbench for 454 and Illumina reads, respectively. A total of 990,111 contigs were then merged to reduce the redundancy by using CAP3, resulting in a total of 503,705 contigs (unpublished data). 454 and Illumina reads have been assembled by CLC Genomic Workbench, MIRA 3 and CAP3 with default parameters.

A preliminary annotation was obtained for 141,001 contigs through Blast similarity searches conducted against several protein databases (e-value 1 E-5, see Table S2).

### Microarray design

All databases used for the annotation step (see Table S2) were considered to reduce the redundancy in annotated contigs. A total of 44,333 contigs found non-redundant (with a unique annotation) in at least one reference database have been considered for microarray design. Putative sense-strand orientation was inferred from the matching protein-coding gene in reference data bases. For 915 contigs that showed ambiguous orientation two probes with opposite orientations (sense and antisense) were designed. For the remaining 43,418 contigs with putatively unambigous orientation a single (sense) probe was designed.

Since the microarray format could accommodate approximately 60,000 probes, non-annotated contigs that showed the highest expression in gonads based on RNA-seq data (see below) were included in the microarray design. In total 7,376 non-annotated contigs were added, and for each of them, two probes with opposite orientation (sense and antisense) were designed. Probe design was carried out using the Agilent eArray interface (https://earray.chem.agilent.com/earray/), which applies proprietary prediction algorithms to design 60-mer probes. A total of 59,951 out of 60,000 probes were successfully obtained, representing 51,709 different *R. decussatus* contigs. The sequences of the 51,709 contigs representing in *R. decussatus* DNA microarray platform have been provided in Table S3. The percentage of annotated transcripts represented on the microarray was 85.7%. In Table S4 has been reported the annotation of each probe represented in DNA microarray platform. Probe sequences and other details on the microarray platform can be found in the GEO database (http://www.ncbi.nlm.nih.gov/geo/) under accession number GPL17766.

### Labeling and microarray hybridization

Sample labeling and hybridization were performed according to the Agilent One-Color Microarray-Based Gene Expression Analysis protocol with the Low Input Quick Amp Labeling kit. Briefly, for each sample 100 ng of total RNA was linearly amplified and labeled with Cy3-dCTP. A mixture of 10 different viral poly-adenylated RNAs (Agilent Spike-In Mix) was added to each RNA sample before amplification and labeling to monitor microarray analysis work-flow. Labeled cRNA was purified with Qiagen RNAeasy Mini Kit, and sample concentration and specific activity (pmol Cy3/μg cRNA) were measured in a NanoDrop® ND-1000 spectrophotometer. A total of 600 ng of labeled cRNA was prepared for fragmentation by adding 5 μl 10X Blocking Agent and 1 μl of 25X Fragmentation Buffer, heated at 60°C for 30 min, and finally diluted by addition with 25 μl 2X GE Hybridization buffer. A volume of 40 μl of hybridization solution was then dispensed in the gasket slide and added to the microarray slide (each slide containing eight arrays). Slides were incubated for 17 h at 65°C in an Agilent hybridization oven, subsequently removed from the hybridization chamber, quickly submerged in GE Wash Buffer 1 to disassemble the slides and then washed in GE Wash Buffer 1 for approximately 1 minute followed by one additional wash in pre-warmed (37°C) GE Wash Buffer 2.

### Data acquisition, correction and normalization

Hybridized slides were scanned at 2 μm resolution using an Agilent G2565BA DNA microarray scanner. Default settings were modified to scan the same slide twice at two different sensitivity levels (XDR Hi 100% and XDR Lo 10%). The two linked images generated were analyzed together and data was extracted and background subtracted using the standard procedures contained in the Agilent Feature Extraction (FE) Software version 10.7.3.1. The software returns a series of spot quality measures in order to evaluate the quality and the reliability of spot intensity estimates. All control features (positive, negative, etc.), except for Spike-in (Spike-in Viral RNAs), were excluded from subsequent analyses.

Raw gene expression data of all 64 analyzed individual gonads (Table 1) were deposited in the GEO database under accession number GSE51150.

Spike-in control intensities were used to identify the best normalization procedure for each dataset. After normalization, spike intensities are expected to be uniform across the experiments of a given dataset. Normalization procedures were performed using R statistical software. Quantile normalization always outperformed cyclic loess and quantile-normalized data were used in all subsequent analyses. Normalized data were deposited in GEO archive under accession number GSE51150.

### Data analysis

Differences in percentage gonad area were analysed using one-way analysis of variance after angular transformation. Comparisons of sex and gametogenic stage distributions between lines were made using Chi-square tests.

A principal component analysis (PCA) using the TMeV 4.5.1 (TIGR MULTIEXPERIMENT VIEWER) [19] was applied to assess the distribution of the studied groups. Statistical tests implemented in the program Significance Analysis of Microarray (SAM) were used to identify differentially expressed probes between the two clam populations. The same approach was used to identify differentially expressed probes in both populations between males and females. A SAM quantitative correlation analysis was also performed to identify genes with a correlation between mRNA levels and gonad area, after angular transformation. A Pavlidis template matching (PTM) analysis was implemented using the TMeV 4.5.1 in order to verify whether the expression of specific genes increased during gonad development. A one-way ANOVA parametric test was used to identify the probes whose expression changed during gonad development in the two populations, using a p-value cut-off of 0.01 and an adjusted Benjamini & Hochberg correction using R software v2.15.2. Hierarchical clustering was performed using TMeV on statistically significant transcripts, to group experimental samples together based on similarity of the overall experimental expression profiling.

A more systematic and functional interpretation for significant gene lists was obtained using an enrichment analysis with the Database for Annotation, Visualisation, and Integrated Discovery (DAVID) software (Huang et al., 2009). “KEGG Pathway”, “Biological process” (BP), “Molecular function” (MF) and “Cellular component” (CC) analyses were carried out by setting the gene count equal to 2 and the ease equal to 0.1. Because the DAVID database contains functional annotation data for a limited number of species, it was necessary to link *R. decussatus* transcripts with sequence identifiers that could be recognized in DAVID. This process was accomplished using dedicated Blast searches performed with Blastx against zebrafish Ensembl proteins. Finally, *Danio rerio* Ensembl Gene IDs were obtained from the corresponding Ensembl protein entries using the BIOMART data mining tool (http://www.ensembl.org/biomart/martview/). *D. rerio* IDs corresponding to differentially transcribed clam genes as well as all of the transcripts that were presented on the array were then used to define a “gene list” and a “background” in DAVID, respectively.

## Results

### Prevalent Gene Expression Patterns

**Figure 3:**
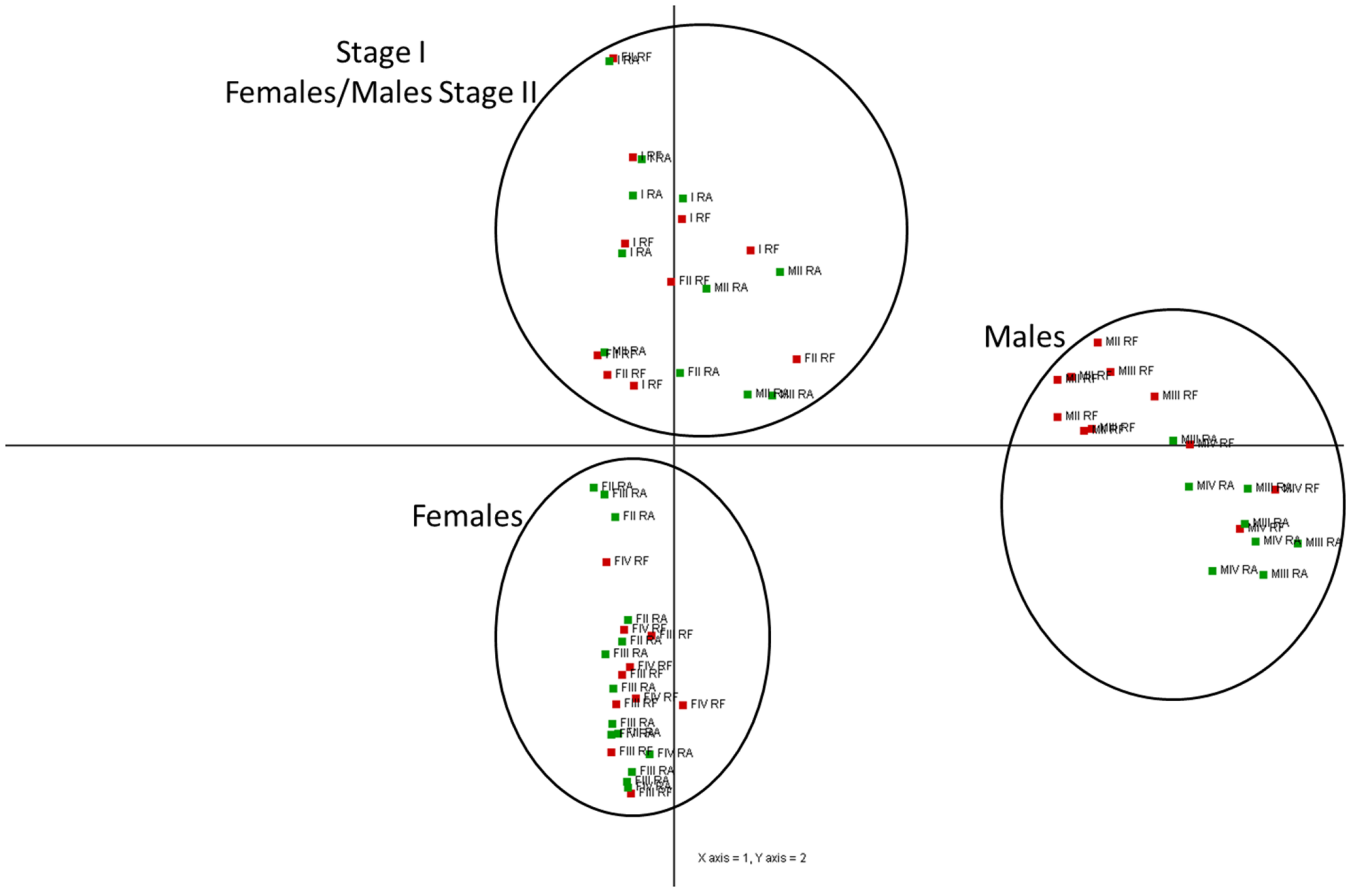
Principal Component Analysis. PCA applied to all 59,951 transcripts of the 64 individual clam gonads sampled from Ria de Aveiro (RA) and Ria Formosa (RF). I =  Stage I (sexual rest); FII =  Female stage II (gametogenesis); FIII =  Female stage III (advanced gametogenesis); FIV =  Female stage IV (maturation); MII =  Male stage II (gametogenesis); MIII =  Male stage III (advanced gametogenesis); MIV =  Male stage IV (maturation).

A Principal Component Analysis (PCA) was applied to the entire gene expression database (59,951 probes) for the 64 clams (Figure 3). We observed a clear clustering of the different sexes and gametogenic stages determined by histological methods. Along the first axis, PC1, which explained 35% of the variation, male and female individuals were discriminated. Gonad developmental stages were organized along the second axis, PC2 (15%). A significant divergence in expression patterns between male and female gonads was observed in stages III and IV (PC2). At these later stages, males and females predictably possess the most distinctive expression profiles, while at stages I and II this separation is not as clear along the second axis.

### Two-unpaired class Significance Analysis of Microarray (SAM) between sexes

In order to compare gene expression profiles between sexes in *R. decussatus*, independently from stage and population of origin, a two-unpaired class Significance Analysis of Microarray (SAM) test was carried out on normalized data (FDR = 5%; FC>1.5). A list of 16,996 significant probes, corresponding to 11,641 unique transcripts, was obtained (Table S5).

**Figure 4:**
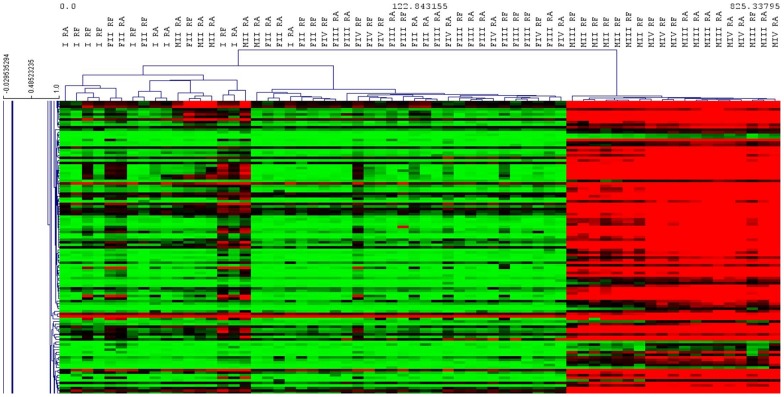
Heat map of sex specific genes. Hierarchical clustering obtained using Pearson's correlation considering the set of 16,996 differentially expressed probes between sexes of the two studied populations of *Ruditapes decussatus*.

Hierarchical clustering using Pearson's correlation for the set of 16,996 differentially expressed probes identified three main clusters: Stage I, Females and Males (Figure 4). However, there are some exceptions to this hierarchical clustering. Indeed, a stage I individual appeared on the same branch as females.

A total of 7,145 differentially expressed probes were up-regulated in the gonads of males with a FC ranging from 1.5 to 255, while a total of 9,751 differentially expressed probes were up-regulated in the females gonads with a FC ranging from 1.5 to 374.

A putative annotation with zebrafish Ensembl Gene IDs was obtained for 6,506 probes that were differentially expressed between the two sexes. These annotated transcripts were used to define a gene list for functional annotation with DAVID. Enrichment analysis showed 9 KEGG, 95 BP terms, 22 CC terms, and 45 MF terms to be significantly over-represented (Table S6).

“Cell cycle” (dre04110), “oocyte meiosis” (04114) and “Progesterone-mediated oocyte maturation” (dre04914) were the most represented in the enriched KEGG pathway terms, with 49, 45 and 38 genes respectively. Cyclin B1 (*CCNB1*) and cyclin B2 (*CCNB2*); Cyclin-Dependent Kinase 2 (CDK2); Mitotic arrest deficient-like 1 (Mad2l1) and several genes of the Calmodulin family (*CaM*) were found among the differentially expressed genes.

The same analysis was performed in each reproductive stage. The number of differentially expressed probes increased as the reproductive stages developed. At stage II a list of 5,059 significant probes, corresponding to 3,237 unique transcripts was obtained. At stage III a list of 18,714 significant probes, corresponding to 12,870 unique transcripts was obtained. Finally, the stage IV represented the highest number of differentially expressed probes with a list of 20,797 probes, corresponding to 14,260 unique transcripts. At this last stage, “oocyte meiosis” (04114) was once again the most represented term for enriched KEGG pathways, with 49 genes. Genes like Speriolin and testis-specific serine/threonine-protein kinase (*TSSK-5*), were present with a high FC value of 332 and 249, respectively, in the list of differentially expressed probes.

### Identification of Stage-specific Expressed Genes

In order to identify those transcripts whose expression changed during gonad development in the two populations, a one-way ANOVA (p<0.01, adjusted Benjamini & Hochberg correction) was performed. This analysis identified 13,218 probes that exhibited a significant change in expression over the reproductive cycle of male and female gonads (Table S7). A putative annotation with *Danio rerio* Ensembl Gene IDs was obtained for 6,451 probes that were differentially expressed between gametogenic stages. Enrichment analysis showed 9 KEGG, 20 BP terms, 12 CC terms, and 40 MF terms to be significantly over-represented (Table S8).

“Focal adhesion” (dre04510) and “Regulation of actin cytoskeleton” (dre04810) were the most represented within enriched KEGG pathway terms, with 74 and 62 genes respectively. “Microtubule-based process” (GO:0007017) was one of the enriched biological processes with the highest representation.

In addition, we decided to emphasize the expression progression of certain well-known reproduction-related genes in the animal kingdom, in order to verify whether their expression increased during gonad development. Vitellogenin (*VTG*), a female specific glycoprotein, was identified in our female samples with an increase in expression along the gametogenic cycle, from stage I to stage IV, with the higher values achieved in stage IV (maturation period). It was also observed that the females from Ria Formosa presented higher expression values of *VTG*. Another female specific gene, *Condensin-2*, was also over-expressed in females during gonad development, with the same trend as *VTG*. In terms of male specific genes, the motile sperm domain containing protein 2 (*MOSPD2*) and the synaptonemal complex protein 2 (*SCP-2*) were identified, showing an increase in expression along the spermatogenesis, from stage I to stage IV.

### Identification of genes with a correlation between mRNA levels and gonad area

Quantitative correlations between microarray data and values of gonad area of stages II, III and IV from males and females, separately, after angular transformation, were evaluated via a SAM quantitative correlation analysis (FDR = 5%). Females showed a total of 10,415 probes significantly (direct or inverse) correlated with the gonad areas (Table S9). Of these probes, 7,226 were annotated by similarity and associated with a known protein. We noticed the presence of positively correlated genes coding for Forkhead box protein L2 (*Foxl2*), *VTG*, *condensing 2*, Mitotic apparatus protein *p62* and *Cep57*. In relation to male gonads, a total of 18,730 probes showed a significant (direct or inverse) correlation with gonad area (Table S10). A putative annotation was obtained for 12,843 probes. Genes expressed included *dpy-30*, sperm associated antigens 6 (*SPAG6*), 16 (*SPAG16*) and 17 (*SPAG17*), *MOSPD2*, sperm surface protein *Sp17* and sperm flagellar proteins 1 and 2. Additionally, functional analysis of significant transcripts showed that different KEGG pathway terms, such as “Cell cycle” (dre04110), “oocyte meiosis” (04114) and “Regulation of actin cytoskeleton” (dre04810), were influenced by gonad area as well.

### Comparison between populations using two-unpaired class Significance Analysis of Microarray (SAM)

In order to compare gene expression between the two sampled populations of *R. decussatus*, considering all stages and sexes, a two-unpaired class Significance Analysis of Microarray (SAM) test was carried out on normalized data, enforcing a False Discovery Rate (FDR) of 5% and a Fold Change threshold of 1.5. A list of 2,900 significant probes, corresponding to 2,228 unique transcripts, was obtained.

A putative annotation with *Danio rerio* Ensembl Gene IDs was obtained for 1,777 probes out of the 2,228 differentially expressed transcripts between the two populations (Table S11). Enrichment analysis revealed a total of 2 KEGG pathways, 23 BP terms, 15 CC terms, and 25 MF terms significantly over-represented among the differentially expressed probes (Table S12). “N-Glycan biosynthesis” (dre00510) term was the most represented in the enriched KEGG pathway terms, with 9 genes.

**Figure 5:**
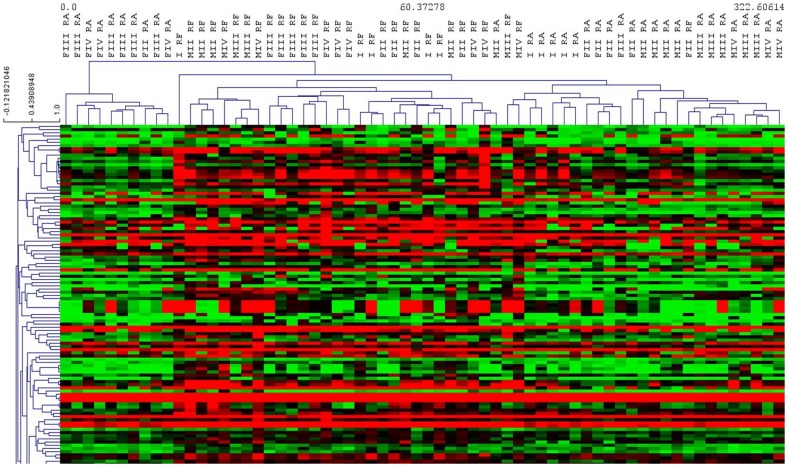
Heat map of population specific genes. Hierarchical clustering obtained using Pearson's correlation considering the set of 2,900 differentially expressed probes between the two studied populations of *Ruditapes decussatus*.

Hierarchical clustering, using Pearson's correlation, was performed considering the set of 2,900 differentially expressed probes. As reported in Figure 5, with some exceptions, three main clusters were identified: Ria Formosa, Ria de Aveiro and Female Stage III/IV from Ria de Aveiro.

In order to support the results obtained by hierarchical clustering, several comparisons between populations in each reproductive stage and sex were performed. The comparison between stage IV females from both populations was the one that presented the highest number of differentially expressed probes. For this analysis, a two-unpaired class Significance Analysis of Microarray (SAM) test was carried out on normalized data (FDR = 5%; FC>1.5). A list of 6,765 significant probes, corresponding to 5,133 unique transcripts, was obtained (Table S13). A putative annotation with *Danio rerio* Ensembl Gene IDs was obtained for 3,229 probes. An enrichment functional annotation analysis was performed with DAVID, revealing 11 KEGG pathways, 88 BP terms, 29 CC terms, and 51 MF terms significantly over-represented (Table S14). “Cell cycle” (dre04110), “Spliceosome” (dre03040), “Ubiquitin mediated proteolysis” (dre04120) and “Oocyte meiosis” (dre04114) terms were the most represented in the enriched KEGG pathway terms, with 45, 32, 30 and 28 genes respectively. Among the genes differentially expressed between females stage IV of the two populations, the great majority were common to “Oocyte meiosis” (dre04114) and “Cell cycle” (dre04110) pathways, like the S-phase kinase-associated protein 1 (*skp1*) and the *Mad2l1*. Other important differentially expressed genes were also identified, such as the *cdc16*, *cdc20*, *cdc27*. Genes like *CCNB1*, cyclin E2 (*CCNE2*), and cdk2 were also present in both pathways.

Another comparison that revealed an important number of differentially expressed probes, at the population level, was between stage II males. A list of 5,801 significant probes, corresponding to 3,588 unique transcripts was obtained (Table S15). A putative annotation with *Danio rerio* Ensembl Gene IDs was obtained for 3,181 probes. An enrichment functional annotation analyses has been performed by DAVID, revealing 14 KEGG pathways, 56 BP terms, 23 CC terms, and 50 MF terms significantly over-represented (Table S16). “Insulin signaling pathway” (dre04910) was the most represented in the enriched KEGG pathway terms. Important differentially expressed genes were found, such as, the Mitogen-Activated Protein Kinase 1 (*MAPK1*). However, for the remaining comparisons performed, no additional significant results were observed between populations.

## Discussion

Recent expression profiling studies using microarrays have been successfully employed in order to better understand the cellular and molecular events of reproductive tissue development and unravel some molecular mechanisms involved in reproductive phenotypes (sex differentiation, gametogenesis; *e.g*. in oyster [5]).

**Figure 6:**
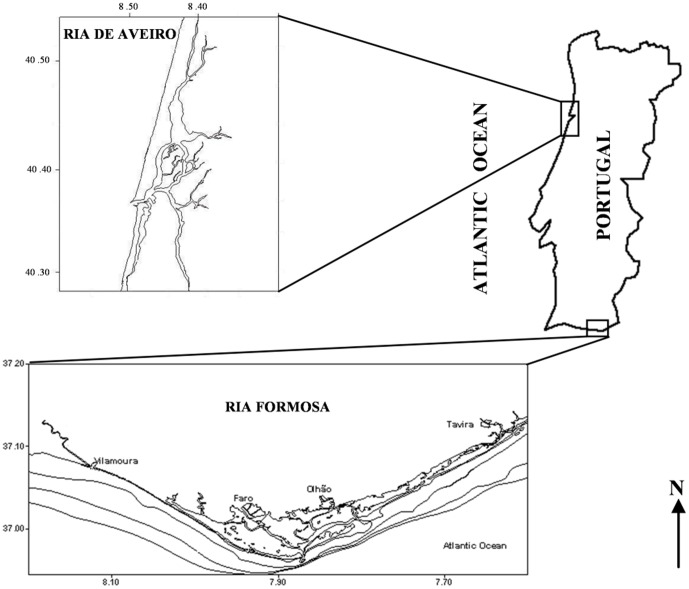
Ria de Aveiro and Ria Formosa Lagoon location. Ria Formosa (37°01′N; 07°49′W) in Southern Portugal and Ria Aveiro (40°42′N; 08°40′W) in Western coast of Portugal.

To better understand the different reproductive behaviors observed between northern and southern Portuguese populations, gonads of clams collected in Ria Formosa (Southern Portugal) and Ria Aveiro (Western coast of Portugal) were analyzed (Figure 6).

### Sex explains most of the variance in gonad gene expression

Although the preliminary analysis using PCA (Figure 3) did not discriminate populations, a clear clustering of the different sexes and gametogenic stages was observed. The main sex effect on gonadic gene expression was also reported for an alternative and irregular protandrous hermaphrodite marine bivalve, the Pacific oyster [5]. The differences between males and females increased over time, following the continuous gametogenesis from stage II to stage IV gonads. Considering gene expression pattern, there is no evidence of sex determination at stage I based on the PCA results, showing that, with this analysis, clam sex is undetermined at stage I.

In the hierarchical clustering performed on the probes differentially expressed between sexes (Figure 4), a stage I individual was detected clustered on the same branch as females. Although the sex of this clam could not be determined histologically, we suggest that this individual had already initiated sex differentiation, being possibly in a later stage I, and could therefore be classified as female according to its gene expression pattern. Indeed, through Pavlidis template matching (PTM) analysis by TMev, we observed that this specific sample over expressed *VTG*, a female specific glycoprotein identified as being necessary for building up the oocyte in oysters [5], among other species. Oysters, at an undetermined sex stage, were found to express male- or female-specific genes, suggesting that sex differentiation already took place at the first stage of gametogenesis, even if it could not be visualized histologically [5]. As a result, in some cases of individuals in later stage I, gene expression patterns could indicate sex determination without histological information.

Following the strong sex effect on gene expression reported above, with 16,996 probes differentially expressed between sexes, 9 KEGG pathways were revealed. Among these pathways, “oocyte meiosis” (dre04114) and “Progesterone-mediated oocyte maturation” (dre04914) were the most represented with 45 and 38 genes, respectively, as well as “Cell cycle” (dre04110) with 49 genes. These pathways are characteristic of females, certainly related to the higher transcription rate in female germ cells compared to male germ cells. Indeed, oocytes provide most of the information for the development of zygotes [20]. Among the genes involved, we found the *Mad2l1*, a component of the spindle-assembly checkpoint that prevents the onset of anaphase until all chromosomes are properly aligned at the metaphase plate, and the *CaM* family, which belongs to one of the two main groups of calcium-binding proteins. Calmodulins are supposed to be very important in the oogenesis of *R. decussatus*, since Ca^2+^ increases are recognized as essential for the oocytes to be released from the metaphase I arrest [21] [22] [23] [24]. The oocytes of the European clam (classified as class II, due to their meiotic maturation distinct from other bivalve species) are held in the ovaries at prophase of the first division of meiosis and their development reinitiates during spawning, by hormonal stimulation or other stimuli. A second barrier to development occurs at metaphase I, prior to extrusion of the first polar body, until fertilization releases this metaphase arrest, allowing further stages of maturation to take place [25]. Furthermore, cyclins, found differentially expressed (*CCNB1*, *CCNB2* and *CDK2*), also trigger metaphase/anaphase transition and further progress through meiosis by disappearing, due to protein synthesis inhibition [25]. In order to better understand the differences between sexes in each reproductive stage, the same analysis was performed in each one. The number of differentially expressed probes increased as the reproductive stages developed, with stage IV presenting the highest number of differentially expressed probes, confirming the results from PCA analysis. Speriolin, a novel centrosomal protein present in the connecting piece region of mouse and human sperm [26], was present in the list of differentially expressed probes between males and females at stage IV, with a FC value of 332, as well as *TSSK-5* with a FC value of 249. mRNA expression of these genes is highly testis-specific, with a very low level detected in other tissues of the marine bivalve *Argopecten purpuratus* [7].

### Differentiation of gonadal developmental stages

From the 13,218 probes that exhibited a significant change in expression over the reproductive cycle of male and female gonads, 9 KEGG pathways appeared notably represented. “Focal adhesion” (dre04510) and “Regulation of actin cytoskeleton” (dre04810), were the most represented, with 74 and 62 genes, respectively. In sea star oocytes, the cortical actin cytoskeleton was noticed to play critical roles in modulating Ca^2+^ release during meiotic maturation, as well as in regulating cortical granule exocytosis [27]. Additionally, “microtubule-based process” (GO:0007017), one of the enriched biological processes with the highest representation, participates in several important events in spermatogenesis, including nuclear elongation, cytoplasmic redistribution and reduction and development of the flagellum [28]. Within the genes involved in this pathway, we found the dynein light chain 2 (*dynll2a* and *dynll2b*), several kinesin family members (*KIF11, KIF17*, and *KIF7*). These genes were also found highly expressed in male gonads in *C. gigas* and may be involved in flagella and cilia structure, locomotion and control [5].

*VTG*, a female specific glycoprotein previously described and *Condensin-2*, expressed in oocytes and involved in maintaining the rigidity of chromosomes in prophase were identified in our female samples with increase in expression as oogenesis progressed. In terms of male specific genes, we identified the *MOSPD2* and the *SCP-2*, involved in the meiotic phase of spermatogenesis, also with an increase in expression during progression of spermatogenesis. All these genes also increased in expression along the gametogenic cycle in male and female gonads of *C. gigas* [5]. *scp2* and *scp3*, genes involved in synaptonemal complex formation, were recently found to be highly expressed in the maturing testis of the scallop *Nodipecten subnodosus* [6]. This synaptonemal complex is a meiosis-specific structure essential for chromosome pairing since disruption of the localization of *scp2* and *scp3* during early recombination resulting in aneuploidy [29]. Our results indicate that synaptonemal complex formation, and therefore meiosis I, was occurring from early to late testis maturation. This results is similar to what was found in *N. subnodosus*, for which the authors concluded that the expression pattern of *scp2* and *scp3* might serve as future markers for the meiotic stage and may prove helpful in differentiating primary and secondary spermatocytes, which is not morphologically possible [6].

A quantitative correlation between microarray data and values of gonad area considered as a proxy of the reproductive effort (*e.g*. in oyster [30]), were evaluated separately for females and males. The clam gonad undergoes continuous development until it becomes fully mature. At that moment, the gonads or gonadal tissue form a significant portion of the soft parts of the animal. Gonaducts that will carry the gametes to the body chamber develop, enlarge and are readily observed in the gonad. For this reason, in males as in females, the gonadal area increases with the evolution of the reproductive stages and is considered as a proxy of the reproductive effort [31]. Reproductive effort and the underlying mechanisms are of great interest for many marine bivalve species that have a very high fecundity, a characteristic of the “r” demographic strategy. Indeed, gametogenesis has a major impact on several physiological functions, generating phenotypic and genetic trade-offs with growth and survival (as established in oysters [32]), meaning that reproductive effort improves at the expense of survival. We identified 7,226 annotated probes correlated with female gonad area and therefore, potentially, with the number and/or the size of the gametes produced. These probes included *Foxl2*, an evolutionarily conserved female-specific transcription factor, which is conserved from the sponge *Suberites domuncula* to humans [33]; *VTG*; *condensing 2*, expressed in oocytes and involved in maintaining the rigidity of chromosomes in prophase; Mitotic apparatus protein *p62* that binds to condensed chromosomes at prophase of meiosis I; and *Cep57*, a centrosomal protein required for microtubule attachment to centrosomes. These female-specific genes, which were positively correlated with gonad areas, were also identified as potentially involved in oogenesis in *C. gigas* [5]. In males, genes expressed included the *SPAG6*, *SPAG16* and *SPAG17*, *MOSPD2*, sperm surface protein *Sp17*, sperm flagellar proteins 1 and 2 and *dpy-30*, all involved in spermatogenesis. *dpy-30* is involved in male development and described as an attractive new candidate gene for the regulation of sex differentiation in oysters [5]. This gene was also identified and characterized as an essential component of the dosage compensation machinery for sex determination in *Caenorhabditis elegans* [34].

### Differential gene expression between populations

In *R. decussatus*, as in other bivalves, the period of spawning in natural populations differs with geographic location and, once reproductive maturity is reached, it may be triggered by several environmental factors including temperature, chemical and physical stimuli, water currents or a combination of these and other factors [14]. The presence of gametes in the water provides an additional stimulus that generates a spawning response in broodstock, due to the presence of hormones (pheromones) that will act as recognition signals between gametes [35]. Indeed, a surface glycoprotein was identified as the mate recognition signal in the caridean shrimp *Palaemonetes pugio* where glucosamine was considered a pheromone functioning in mate recognition [36]. Although it had a low statistical significance value for enrichment in DAVID (P = 0.09), “N-Glycan biosynthesis” (dre00510) was found among the enriched KEGG pathways with the highest number of gene entries. N-glycans have an important role acting as recognition signals and binding between the extracellular coat of the egg and components of the sperm plasma membrane, generally termed “primary binding” [37]. Taking into account that “N-Glycan biosynthesis” (dre00510) pathway was down-regulated in the population of Ria Formosa, we propose that, in some way, this recognition process can explain some of the differences in spawning induction success observed between the two Portuguese populations. Indeed, it was previously demonstrated that environment can strongly affect reproduction of *R. decussatus*, notably in terms of fecundity level [14]. Additionally, among the genes involved in N-Glycan biosynthesis, we found fucosyltransferase (*FucT*) differentially expressed with a fold change of 2.23. This enzyme is implicated in species-specific binding of sperm to eggs in mammals [38] [39] [40]. In addition, ADAMs family genes (A Disintegrin and A Metalloprotease domain) as well as several integrin ligands appeared differentially expressed between the two populations. Several studies have indicated that these molecules are involved in cell adhesion and sperm-egg interaction (*e.g*. [41] [42] [43]), supporting the hypothesis that “primary binding” could be responsible for the main differences in the reproductive behavior of the two populations.

Recent histological examinations of these two populations showed significant differences in the dynamics of gametogenesis [14]: the maturation process begins earlier in the northern population probably due to contrasted environmental factors mainly temperature, which is already characterized as the main source of variation in the timing of gametogenesis (*e.g*. [18]). Sea surface temperature (SST) was relatively lower compared to Ria Formosa lagoon, though with higher chlorophyll values [14]. Nonetheless, similar values of oocyte diameter and gonadic area were reported, suggesting similar investment in reproduction in the two populations. In terms of gene expression, hierarchical clustering using Pearson's correlation, identified three main clusters: Ria Formosa, Ria de Aveiro and Female Stage III and IV from Ria de Aveiro (Figure 5), suggesting that the biggest differences originate from the females in the later stages of gametogenesis, and may be due to a difference in gamete maturity that may be implicated in the differences in response to spawning induction. These differences can originate from environmental differences and/or some genetically based variation between the two populations. Among all the comparisons made between populations for each reproductive stage and sex, the one performed with stage IV females presented the highest number of differentially expressed probes (5,133 probes; Table S13). In this comparison, “Cell cycle” (dre04110), “Spliceosome” (dre03040), “Ubiquitin mediated proteolysis” (dre04120) and “Oocyte meiosis” (dre04114) terms were the most represented in the enriched KEGG pathway terms with 45, 32, 30 and 28 genes, respectively. Due to their specific environments, these populations might adopt different reproductive strategies: the Ria de Aveiro population retrieves its energy reserves immediately after spawning, however, the same is not verified in clams from Ria Formosa Lagoon, with their consequent decline. Considering these facts, it is likely that, at some point, the process of oogenesis diverged between females of the two populations. Among the genes differentially expressed, the *SKP1* and the *Mad2l1* previously described, were present. The *SKP1* is an essential component of the SCF (SKP1-CUL1-F-box protein) ubiquitin ligase complex, which mediates the ubiquitination of proteins involved in cell cycle progression, signal transduction and transcription. Other important differentially expressed genes were found, such as *cdc16*, *cdc20*, *cdc27*, which are components of the anaphase promoting complex/cyclosome (APC/C), a cell cycle-regulated E3 ubiquitin ligase controlling progression through mitosis and the G1 phase of the cell cycle. In addition, genes like *CCNB1* and *CCNE2* were present and are essential in controlling the progression of cells through the cell cycle, by activating *Cdk* enzymes. Cyclin-encoding transcripts represent maternal mRNAs, known to be stocked in oocytes during oogenesis and maternally transmitted to embryos before the start of embryonic transcription [44].

A total of 3,588 differentially expressed probes were also obtained in the comparison made between males of stage II. “Insulin signaling pathway” (dre04910) was the most represented in the enriched KEGG pathway terms. In addition to its function in somatic cells, it was demonstrated that insulin signaling plays an essential role in cell proliferation and growth during male *Drosophila* gametogenesis where sperm production is regulated by hormonal control via insulin-like peptides [45]. Moreover, the level of some mRNA in insulin pathway was also identified as correlated with gamete quality in fishes [46] [47]. Additionally, the *MAPK1* was found differentially expressed. Studies have shown that male reproductive function is modulated via the *MAPK* cascade. The *MAPK* cascade is involved in numerous male reproductive processes, including spermatogenesis, sperm maturation and activation, capacitation and acrosome reaction, before fertilization of the oocyte [48]. Considering that no additional significant results were observed for the remaining comparisons performed, we suggest that it is in the later stages of oogenesis (stage IV) and in the earlier stages of spermatogenesis (stage II), that the major differences between the two studied populations occur.

## Conclusions

In this study, we identified several differentially expressed probes, which led us to a specific pathway involved in the recognition signals and binding between the oocyte and components of the sperm plasma membrane. We believe that these results can be useful to *R. decussatus* hatchery production program management. Furthermore, as a possible starting point for further research devoted to reproduction and spawning improvement of this species, we identified genes specifically expressed in either males or females that showed increasing expression over the course of gametogenesis.

The designed custom oligonucleotide microarray can be useful for future studies into the molecular mechanisms involved in the European clam differentiation and development. Additionally, we produced lists of relevant pathways and candidate genes that helped to improve our understanding of gametogenesis and that are essential in addressing physiological, environmental or aquaculture questions concerning the European clam *R. decussatus*.

## Supporting Information

Table S1 **Summary of the origin of** ***Ruditapes decussatus*** **sequences and assembly information.**
(XLSX)

Table S2 **List of protein database used for contigs annotation obtained through Blast similarity searches.** Numbers of annotated contigs for each database are also reported.
(XLSX)

Table S3 **Sequences of the 51,709 different contigs of** ***R. decussatus*** **representing in the DNA microarray platform.**
(ZIP)

Table S4 **List and annotation of each probe represented in the** ***R. decussatus*** **DNA microarray platform.**
(XLSX)

Table S5 **List of significant probes identified by SAM analysis (FDR = 5%; FC>1.5) by comparing sexes in** ***Ruditapes decussatus*** **.**
(XLSX)

Table S6 **Enrichment analysis showing KEGG pathways, BP terms, CC terms and MF terms significantly represented on differentially expressed probes between sexes in** ***Ruditapes decussatus*** **.**
(XLSX)

Table S7 **List of significant probes identified by One-way ANOVA (p<0.01, adjusted Benjamini & Hochberg correction) in order to identify the transcripts whose expression changed over the reproductive cycle of male and female gonads.**
(XLSX)

Table S8 **Enrichment analysis showing KEGG pathways, BP terms, CC terms and MF terms significantly represented on differentially expressed probes between gametogenic stages in** ***Ruditapes decussatus*** **.**
(XLSX)

Table S9 **List of significant probes identified by SAM quantitative correlation analysis (FDR = 5%) between microarray data and values of female gonad area of stages II, III and IV after angular transformation in** ***Ruditapes decussatus*** **.**
(XLSX)

Table S10 **List of significant probes identified by SAM quantitative correlation analysis (FDR = 5%) between microarray data and values of male gonad area of stages II, III and IV after angular transformation in** ***Ruditapes decussatus*** **.**
(XLSX)

Table S11 **List of significant probes identified by SAM analysis (FDR = 5%; FC>1.5) by comparing the two populations of** ***Ruditapes decussatus*** **.**
(XLSX)

Table S12 **Enrichment analysis showing KEGG pathways, BP terms, CC terms and MF terms significantly represented on differentially expressed probes between populations of** ***Ruditapes decussatus*** **.**
(XLSX)

Table S13 **List of significant probes identified by SAM analysis (FDR = 5%; FC>1.5) by comparing stage IV females from both populations of** ***Ruditapes decussatus*** **.**
(XLSX)

Table S14 **Enrichment analysis showing KEGG pathways, BP terms, CC terms and MF terms significantly represented on differentially expressed probes between stage IV females from both populations of** ***Ruditapes decussatus*** **.**
(XLSX)

Table S15 **List of significant probes identified by SAM analysis (FDR = 5%; FC>1.5) by comparing stage II males from both populations of** ***Ruditapes decussatus*** **.**
(XLSX)

Table S16 **Enrichment analysis showing KEGG pathways, BP terms, CC terms and MF terms significantly represented on differentially expressed probes between stage II males from both populations of** ***Ruditapes decussatus*** **.**
(XLSX)

## References

[1] FAO (2006). Available: ftp://fao.org/fi/stat/summary/a-6.pdf.

[2] Matias D, Joaquim S, Leitao A, Massapina C (2009) Effect of geographic origin, temperature and timing of broodstock collection on conditioning, spawning success and larval viability of *Ruditapes decussatus* (Linn, 1758). Aquac Int 17: 257–271.

[3] Vilela H (1950) Vida bentonica de *Tapes decussatus* (L.). Travail Station de Biologie Marine Lisbonne 53, 1–79 (In portuguese).

[4] Delgado M, Pérez Camacho A (2005) Histological study of the gonadal development of *Ruditapes decussatus* (L.) (Mollusca: Bivalvia) and its relationship with available food. Sci Mar 69 (1): 87–97.

[5] Dheilly NM, Lelong C, Huvet A, Kellner K, Dubos M-P, et al (2012) Gametogenesis in the Pacific Oyster *Crassostrea gigas*: A Microarrays-Based Analysis Identifies Sex and Stage Specific Genes. PLoS ONE 7: e36353. 22590533 10.1371/journal.pone.0036353 PMC3348941

[6] Llera-Herrera R, García-Gasca A, Abreu-Goodger C, Huvet A, Ibarra AM (2013) Identification of Male Gametogenesis Expressed Genes from the Scallop *Nodipecten subnodosus* by Suppressive Subtraction Hybridization and Pyrosequencing. PLoS ONE 8: e73176. 24066034 10.1371/journal.pone.0073176 PMC3774672

[7] Boutet I, Moraga D, Marinovic L, Obreque J, Chavez-Crooker P (2008) Characterization of reproduction-specific genes in a marine bivalve mollusc: Influence of maturation stage and sex on mRNA expression. Gene 407: 130–138. 17976928 10.1016/j.gene.2007.10.005

[8] Fabioux C, Huvet A, Lelong C, Robert R, Pouvreau S, et al (2004) Oyster vasa-like gene as a marker of the germline cell development in *Crassostrea gigas* . Biochem Biophys Res Commun 320: 592–598. 15219870 10.1016/j.bbrc.2004.06.009

[9] Fabioux C, Pouvreau S, Le Roux F, Huvet A (2004) The oyster vasa-like gene: a specific marker of the germline in *Crassostrea gigas* . Biochem Biophys Res Commun 315: 897–904. 14985097 10.1016/j.bbrc.2004.01.145

[10] Fleury E, Fabioux C, Lelong C, Favrel P, Huvet A (2008) Characterization of a gonad-specific transforming growth factor-beta superfamily member differentially expressed during the reproductive cycle of the oyster *Crassostrea gigas* . Gene 410: 187–196. 18234456 10.1016/j.gene.2007.12.017

[11] Obata M, Sano N, Kimata S, Nagasawa K, Yoshizaki G, et al (2010) The proliferation and migration of immature germ cells in the mussel, *Mytilus galloprovincialis*: observation of the expression pattern in the M. galloprovincialis vasa-like gene (Myvlg) by in situ hybridization. Dev Genes Evol 220: 139–149. 20725841 10.1007/s00427-010-0335-3

[12] Moreira R, Balseiro P, Romero A, Dios S, Posada D, et al (2012) Gene expression analysis of clams *Ruditapes philippinarum* and *Ruditapes decussatus* following bacterial infection yields molecular insights into pathogen resistance and immunity. Dev Comp Immunol 36: 140–149. 21756933 10.1016/j.dci.2011.06.012

[13] Leite RB, Milan M, Coppe A, Bortoluzzi S, Anjos A dos, et al (2013) mRNA-Seq and microarray development for the Grooved carpet shell clam, *Ruditapes decussatus*: a functional approach to unravel host -parasite interaction. BMC Genomics 14: 741. 24168212 10.1186/1471-2164-14-741 PMC4007648

[14] Matias D, Joaquim S, Matias AM, Moura P, de Sousa JT, et al (2013) The reproductive cycle of the European clam *Ruditapes decussatus* (L., 1758) in two Portuguese populations: Implications for management and aquaculture programs. Aquaculture 406–407: 52–61.

[15] Guillard RRL (1975) Culture of Phytoplankton for Feeding Marine Invertebrates. In: Smith WL, Chanley MH, editors.Culture of Marine Invertebrate Animals. Springer US. pp. 29–60.

[16] Latendresse JR, Warbrittion AR, Jonassen H, Creasy DM (2002) Fixation of testes and eyes using a modified Davidson's fluid: comparison with Bouin's fluid and conventional Davidson's fluid. Toxicol Pathol 30: 524–533. 12187944 10.1080/01926230290105721

[17] Martoja R, Martoja-Pierson M (1967) Initiation aux techniques de l'histologie animale. Elsevier Masson. 345 p.

[18] Fabioux C, Huvet A, Le Souchu P, Le Pennec M, Pouvreau S (2005) Temperature and photoperiod drive *Crassostrea gigas* reproductive internal clock. Aquaculture 250: 458–470.

[19] Saeed AI, Sharov V, White J, Li J, Liang W, et al (2003) TM4: a free, open-source system for microarray data management and analysis. BioTechniques 34: 374–378. 12613259 10.2144/03342mt01

[20] Watson AJ (2007) Oocyte cytoplasmic maturation: A key mediator of oocyte and embryo developmental competence. J Anim Sci 85: E1–E3. 10.2527/jas.2006-432 17322120

[21] Hidaka H (1985) Calmodulin antagonists and cellular physiology. Elsevier. 569 p.

[22] Gobet I, Durocher Y, Leclerc C, Moreau M, Guerrier P (1994) Reception and Transduction of the Serotonin Signal Responsible for Meiosis Reinitiation in Oocytes of the Japanese Clam *Ruditapes philippinarum* . Dev Biol 164: 540–549. 8045350 10.1006/dbio.1994.1222

[23] Deguchi R, Morisawa M (2003) External Ca2+ is predominantly used for cytoplasmic and nuclear Ca2+ increases in fertilized oocytes of the marine bivalve *Mactra chinensis* . J Cell Sci 116: 367–376. 12482922 10.1242/jcs.00221

[24] Tosti E (2006) Calcium ion currents mediating oocyte maturation events. Reprod Biol Endocrinol 4: 26. 16684344 10.1186/1477-7827-4-26 PMC1475868

[25] Colas P, Dubé F (1998) Meiotic maturation in mollusc oocytes. Semin Cell Dev Biol 9: 539–548. 9835642 10.1006/scdb.1998.0248

[26] Goto M, O'Brien DA, Eddy EM (2010) Speriolin is a novel human and mouse sperm centrosome protein. Hum Reprod Oxf Engl 25: 1884–1894. 10.1093/humrep/deq138 PMC2907228 20542897

[27] Kyozuka K, Chun JT, Puppo A, Gragnaniello G, Garante E, et al (2008) Actin cytoskeleton modulates calcium signaling during maturation of starfish oocytes. Dev Biol 320: 426–435. 18599031 10.1016/j.ydbio.2008.05.549

[28] Sperry AO (2012) The dynamic cytoskeleton of the developing male germ cell. Biol Cell 104: 297–305. 22276751 10.1111/boc.201100102 PMC3845902

[29] Shin Y-H, Choi Y, Erdin SU, Yatsenko SA, Kloc M, et al (2010) Hormad1 Mutation Disrupts Synaptonemal Complex Formation, Recombination, and Chromosome Segregation in Mammalian Meiosis. PLoS Genet 6: e1001190. 21079677 10.1371/journal.pgen.1001190 PMC2973818

[30] Royer J, Seguineau C, Park K-I, Pouvreau S, Choi K-S, et al (2008) Gametogenetic cycle and reproductive effort assessed by two methods in 3 age classes of Pacific oysters, *Crassostrea gigas*, reared in Normandy. Aquaculture 277: 313–320.

[31] Serdar S, Lok A (2010) Monthly Variations in Gonadal Development and Biochemical Composition of the Carpet Shell Clam Ruditapes (Tapes) Decussatus, Linnaeus 1758 in Mersin Bay, Aegean Sea, Turkey. Fresenius Environ Bull 19: 1055–1063.

[32] Ernande B, Boudry P, Clobert J, Haure J (2004) Plasticity in resource allocation based life history traits in the Pacific oyster, *Crassostrea gigas*. I. Spatial variation in food abundance. J Evol Biol 17: 342–356. 15009268 10.1046/j.1420-9101.2003.00674.x

[33] Lee K, Pisarska MD, Ko J-J, Kang Y, Yoon S, et al (2005) Transcriptional factor FOXL2 interacts with DP103 and induces apoptosis. Biochem Biophys Res Commun 336: 876–881. 16153597 10.1016/j.bbrc.2005.08.184

[34] Hsu DR, Meyer BJ (1994) The dpy-30 gene encodes an essential component of the *Caenorhabditis elegans* dosage compensation machinery. Genetics 137: 999–1018. 7982580 10.1093/genetics/137.4.999 PMC1206076

[35] Parwadani Aji L (2011) Review: Spawning Induction in Bivalve. J Penelit Sains 14: 33–36.

[36] Caskey JL, Hasenstein KH, Bauer RT (2009) Studies on contact sex pheromones of the caridean shrimp *Palaemonetes pugio*: I. Cuticular hydrocarbons associated with mate recognition. Invertebr Reprod Dev 53: 93–103.

[37] Di Patrizi L, Capone A, Focarelli R, Rosati F, Gallego R, et al (2001) Structural characterization of the N-glycans of gp273, the ligand for sperm-egg interaction in the mollusc bivalve *Unio elongatulus* . Glycoconj J 18: 511–518. 12151712 10.1023/a:1019617728660

[38] Wassarman PM, Jovine L, Litscher ES (2001) A profile of fertilization in mammals. Nat Cell Biol 3: E59–E64. 11175768 10.1038/35055178

[39] Chiu PCN, Chung M-K, Koistinen R, Koistinen H, Seppala M, et al (2007) Glycodelin-A interacts with fucosyltransferase on human sperm plasma membrane to inhibit spermatozoa-zona pellucida binding. J Cell Sci 120: 33–44. 17148576 10.1242/jcs.03258

[40] Venditti JJ, Swann JM, Bean BS (2010) Hamster Sperm-Associated Alpha-l-Fucosidase Functions During Fertilization. Biol Reprod 82: 572–579. 19794153 10.1095/biolreprod.109.076695

[41] Evans JP (2001) Fertilin beta and other ADAMs as integrin ligands: insights into cell adhesion and fertilization. BioEssays News Rev Mol Cell Dev Biol 23: 628–639. 10.1002/bies.1088 11462216

[42] Fard Jahromi SS, Shamsir MS (2013) Construction and Analysis of the Cell Surface's Protein Network for Human Sperm-Egg Interaction. ISRN Bioinforma 2013: 1–8. 10.1155/2013/962760 PMC4393059 25937952

[43] Eto K, Huet C, Tarui T, Kupriyanov S, Liu H-Z, et al (2002) Functional classification of ADAMs based on a conserved motif for binding to integrin alpha 9beta 1: implications for sperm-egg binding and other cell interactions. J Biol Chem 277: 17804–17810. 11882657 10.1074/jbc.M200086200

[44] Wada T, Hara M, Taneda T, Qingfu C, Takata R, et al. (2012) Antisense morpholino targeting just upstream from a poly(A) tail junction of maternal mRNA removes the tail and inhibits translation. Nucleic Acids Res 40.. 10.1093/nar/gks765 PMC3526265 22904086

[45] Ueishi S, Shimizu H, Inoue YH (2009) Male Germline Stem Cell Division and Spermatocyte Growth Require Insulin Signaling in *Drosophila* . Cell Struct Funct 34: 61–69. 19384053 10.1247/csf.08042

[46] Aegerter S, Jalabert B, Bobe J (2004) Messenger RNA stockpile of cyclin B, insulin-like growth factor I, insulin-like growth factor II, insulin-like growth factor receptor Ib, and p53 in the rainbow trout oocyte in relation with developmental competence. Mol Reprod Dev 67: 127–135. 14694427 10.1002/mrd.10384

[47] Bobe J, Maugars G, Nguyen T, Rime H, Jalabert B (2003) Rainbow trout follicular maturational competence acquisition is associated with an increased expression of follicle stimulating hormone receptor and insulin-like growth factor 2 messenger RNAs. Mol Reprod Dev 66: 46–53. 12874798 10.1002/mrd.10334

[48] Li MWM, Mruk DD, Cheng CY (2009) Mitogen-activated protein kinases in male reproductive function. Trends Mol Med 15: 159–168. 19303360 10.1016/j.molmed.2009.02.002 PMC2804913

